# Effects of Excessive Screen Time on Child Development: An Updated Review and Strategies for Management

**DOI:** 10.7759/cureus.40608

**Published:** 2023-06-18

**Authors:** Sudheer Kumar Muppalla, Sravya Vuppalapati, Apeksha Reddy Pulliahgaru, Himabindu Sreenivasulu

**Affiliations:** 1 Pediatrics, PES (People's Education Society) Institute of Medical Sciences and Research, Kuppam, IND; 2 General Practice, PES (People's Education Society) Institute of Medical Sciences and Research, Kuppam, IND; 3 Pediatric Medicine, PES (People's Education Society) Institute of Medical Sciences and Research, Kuppam, IND; 4 General Medicine, PES (People's Education Society) Institute of Medical Sciences and Research, Kuppam, IND

**Keywords:** campaign, developmental and behavioral delay, digital learning, parenting style, screen exposure time, screen time, language development, cognitive development

## Abstract

Children's heavy reliance on screen media has raised serious public health issues since it might harm their cognitive, linguistic, and social-emotional growth. This study examines the effects of screen time on many developmental domains and covers management and limitation techniques for kids' screen usage. Screen media has a wide range of cognitive consequences, with both beneficial and detrimental effects noted. Screens can improve education and learning; however, too much time spent in front of a screen and multitasking with other media has been related to worse executive functioning and academic performance. As screen time reduces the amount and quality of interactions between children and their caregivers, it can also have an impact on language development. Contextual elements like co-viewing and topic appropriateness are key in determining how language development is impacted. Additionally, excessive screen usage has detrimental effects on social and emotional growth, including a rise in the likelihood of obesity, sleep disorders, and mental health conditions including depression and anxiety. It can obstruct the ability to interpret emotions, fuel aggressive conduct, and harm one's psychological health in general. Setting boundaries, utilizing parental controls, and demonstrating good screen behavior are all techniques that parents may use to manage children's screen usage. We can reduce the possible negative impacts of excessive screen time and promote children's healthy development and well-being by increasing knowledge and encouraging alternative activities that stimulate development.

## Introduction and background

New technologies, such as mobile and interactive screen media, are now ingrained in a young child's daily life. Children today are "digital natives," having been born into an ever-changing digital ecosystem augmented by mobile media. The age at which kids engage with media on a regular basis has fallen from four years in 1970 to four months in the present day [1]. Electronic devices have revolutionized learning, communication, and information dissemination, but recent research indicates that screen media use may have serious adverse effects on children's health over the long term, making this a pressing public health concern [2]. It has raised the likelihood that children will become obese, experience behavioral problems, sleep irregularities, poor academic performance, etc. [1-3].

This article delves into the profound effects of excessive screen time on children's cognitive, language, and social-emotional development, while also exploring the crucial strategies and responsibilities that parents and schools can undertake to effectively manage and diminish screen time in young individuals.

## Review

Impact on development

Cognitive Development

Screen media use may have both beneficial and detrimental effects on a kid’s cognitive results. Media devices with screens have the potential to improve education and learning [2]. For instance, research has suggested that electronic books and learning-to-read applications may improve young children's early reading skills and creative thinking capacities [2,4,5]. However, studies have also demonstrated the negative effects of screen media use on a number of cognitive areas such as executive functioning, sensorimotor development, and academic outcomes [2,6]. Media multitasking was found to have a negative impact on executive functioning in teenagers, notably on working memory, inhibition, and the capacity to switch between tasks [2,7].

The Quebec Longitudinal Study of Child Development cohort study found a long-lasting connection between early screen media exposure and cognitive abilities, with each one-hour increase in TV exposure at two years of age corresponding to a 7% unit decrease in participation in class and a 6% unit decrease in math proficiency in the fourth grade [8]. A Spanish research study discovered a negative correlation between the use of screen media and academic achievement, indicating that increased screen time was associated with lower academic performance. Similarly, a study conducted in the United States found a significant link between higher levels of media multitasking and lower scores on standardized tests measuring academic performance in mathematics and English [9].

However, adverse executive and academic outcomes may be confounded by the poorer attention and focus from multitasking behaviors rather than solely from screen media overuse, and more research is necessary to tease out this relationship. Language development has received the majority of emphasis in studies on screen usage and cognitive development in young children, whereas other cognitive development domains including executive skills have received less attention [10]. Thus, there are significant, albeit indirect, effects of screen media use on cognitive development in children [2].

Language Development​​

The early years of childhood are crucial for acquiring language skills and children develop various aspects of language, including vocabulary and phonology [11]. These skills are acquired through interactions with adults [11]. Numerous studies have highlighted the significance of human interaction, particularly the frequency and quality of exchanges between adults and children, in the development of language skills [12]. However, there is a growing concern that screen time diminishes the quantity and quality of interactions between children and their parents, resulting in fewer chances for the child to practice and develop their language abilities [11]. The relationship between screen time and speech and language development is complex, and there are multiple factors that should be taken into account [13]. The impact of screen viewing is predominantly influenced by contextual factors rather than the sheer amount of time spent watching [14]. The context encompasses several aspects, such as the behavior exhibited by adult caregivers during screen time, the appropriateness of the content for the child's age, and the level of interactivity provided by the screen [14]. Increasing the amount of screen time at an early age has negative effects on language development [13]. However, beginning screen time at a later age has some potential benefits [13]. The characteristics of videos, their content, and co-viewing also play a role in influencing language development [13]. However, other studies have reported negative effects on speech, language, motor skills, cognitive development, and social development [13].

Adults should be aware of the impact of background television when children are present [13]. Studies have shown that increased exposure to background television can have adverse effects on children's language usage, executive functioning, and cognition in children under the age of five [13]. Excessive television viewing can also potentially affect language development and reading abilities at a young age [13].

A few studies show a positive correlation between children's screen time when co-viewed with a parent and their expressive lexical, phonological, and overall language abilities [11]. Studies have indicated that compared to children who view screens for ≤1 hour per day, those who engage in two or more hours per day, or three or more hours per day, are more likely to experience behavioral problems and have poorer vocabulary acquisition [15]. These findings suggest the importance of monitoring and regulating screen time for young children to mitigate potential adverse effects on their development and behavior [15].

While it is true that screens have become ubiquitous in homes and are increasingly integrated into school systems, it is crucial to educate caregivers of children under the age of three years about the potential risks associated with prolonged screen exposure in inappropriate contexts [14]. The current findings suggest that future research should investigate the screen time patterns of the entire family and explore the cumulative impact of both children's and parents' total screen time on language skills, specifically focusing on lexical skills [11]. In addition to providing guidance, it is crucial to offer families alternative options to media use that promote positive development, such as parent-child play activities [16]. These alternatives should be made available in pediatric practices and early childhood centers to support families in creating enriching and interactive experiences with their children [16].

Social-Emotional Development

In recent years, the concept of screen time has become more complex, with a wide range of electronic devices available worldwide [17]. The advancement of technology in recent years has led to increased screen-based technology usage among young people, coinciding with a decrease in their engagement with nature and impacting their mental health and overall well-being [18]. Research has shown negative associations between screen time, particularly television viewing, and the development of physical and cognitive abilities. Additionally, screen time has been linked to obesity, sleep problems, depression, and anxiety [17]. The specific physiological mechanisms underlying the adverse health outcomes related to screen time, as well as the relative contributions of different types of screens and media content to these outcomes, are still not fully understood [17]. However, studies in very young children indicate that screen usage is an independent risk factor for reduced psychological well-being. One study shows that increased TV exposure between six and 18 months of age was associated with emotional reactivity, aggression, and externalizing behaviors [17]. Few studies indicate that higher screen time at the age of four years is associated with lower levels of emotional understanding at the age of six years. It also reveals that having a television in a child's bedroom at the age of six years predicts lower levels of emotional understanding at eight years [19]. Gaming was associated with lower levels of emotional understanding in boys but not in girls. This suggests that different types of screen activities may have distinct effects on the emotional development of children based on their gender [19].

Computer use and video gaming, but not television viewing, were shown to be connected with more severe depressive symptoms when looking at the effects of various types of screens. Video gaming, in particular, is correlated with the severity of anxiety [17]. These findings align with other studies showing a cumulative impact of high screen time on symptoms, with more pronounced effects emerging during early adolescence and beyond [17]. Screen time-induced poor sleep, nighttime use of digital devices, and dependency on mobile phones have been associated with depressive symptoms [20]. Sleep issues, excessive screen time, and exposure to content that is violent and fast-paced trigger dopamine and reward pathways in the brain, all of which have been associated with attention-deficit/hyperactivity disorder (ADHD)-related behavior [20]. Early and persistent exposure to violent content raises the chance of engaging in antisocial behavior [20]. Psychoneurological effects of addictive screen time use include a decrease in social coping skills and the development of craving behaviors resembling substance dependence [20]. Structural changes in the brain related to cognitive control and emotional regulation have been observed in individuals with addictive digital media behavior [20].

It is worth noting that screens can also have positive educational and informational benefits [17]. For example, many schools are effectively using blogs as educational tools to improve written English, and the Internet provides access to health resources, including information on sexually transmitted infections and mental health [17]. Starting around the age of two years, high-quality television programs that are designed for specific educational purposes can serve as an additional means for early language and literacy development in children [21]. Such programs can also support cognitive development, promote positive racial attitudes, and encourage imaginative play [21]. High-quality content can enhance social and language skills for all children aged two years and older, particularly for those who are living in poverty or facing other disadvantages [21]. The presence of smartphones blurs the boundaries between work and home life, making timing unpredictable, and frequently requiring emotional investment to respond to them [21]. These findings emphasize the potential negative consequences of excessive screen exposure during early childhood, particularly when screens are present in a child's personal space, such as their bedroom [19]. This study emphasizes the significance of face-to-face interaction, especially with primary caregivers, in promoting the development of social-emotional competence in young children [19]. Further research is needed to investigate the mechanisms underlying the connection between screen time and developmental vulnerabilities [22].

Strategies for managing and reducing screen time in children

Strong evidence shows that parents' awareness-raising and other straightforward actions may significantly lower children's screen time [23]. Early excessive screen-watching habits seem to follow throughout time and tend to cluster with other harmful lifestyle behaviors including a poor diet and lack of sleep [24]. The amount of time spent watching television and other forms of screen time among teens decreased only when the intervention included specific elements or activities aimed at reducing it [25]. Possible additions to interventions include the use of an electronic monitoring device to restrict screen time (which allows users to set time limits for TV, digital versatile disc (DVD), computer, or video game use), the TV Turn-off Challenge (a campaign to turn off the TV for a specified number of days), the conditional use of screens on physical activity, or education via mass or small media (such as newsletters, brochures, or billboards) [26]. Birth kits distributed to moms by maternity wards should contain information regarding newborns and toddlers seeing screens [23]. Health visitors should provide new parents with advice and be aware of medical evidence [23]. Schools should take a stand on how much time kids spend using screens both inside and outside of the classroom and let students and parents know about it [23].

Role of Parents in Managing Screen Time

Parents sometimes used screen time as a reward but also believed digital technology would have a negative impact on their child’s behavior, social skills, sleep, and physical activity [27]. Parents have the chance to implement behavioral control in the home as the primary caretakers, frequently through observation and rule-setting [27]. In fact, treatments designed to enhance common parenting techniques at home have proved effective in raising a child's desirable behavior [27]. In line with this viewpoint, some research indicates that parents' usage of restrictions regarding the use of technology such as television, cellphones, tablets, and computers is linked to children spending less time on screens (such as television, video games, and computer/internet use) [28]. Parental controls, which are frequently present in the form of extra settings and password safeguards for different technical devices (such as televisions, laptops, cellphones, etc.), may offer a viable answer to parenting challenges around children's screen time [27].

The Centers for Disease Control and Prevention (CDC) and other organizations/studies have indicated that parental restrictions on screen time and the absence of screens in bedrooms both significantly lower screen time [29,30]. Ideal discretionary screen time limits are 0.5-1 hour/day for three to seven-year-olds, one hour for 7-12-year-olds, 1.5 hours for 12-15-year-olds, and two hours for 16+-year-olds. Role modeling is also another crucial element. The amount of screen time parents and kids watch is closely associated; kids who live in homes where watching TV is encouraged (e.g., meals eaten in front of the TV and the TV is on when the child gets home from school) are more likely to engage in binge-watching themselves. If parents watch television for more than four hours every day, their sons and daughters will, respectively, have a 10.5-fold and a three-fold increased likelihood of doing the same [31].

## Conclusions

Excessive screen media usage in children can have both positive and negative impacts on their development. Regarding cognitive development, screens have the potential to enhance education and learning. However, studies have shown that excessive screen time and media multitasking can negatively affect executive functioning, sensorimotor development, and academic outcomes. Early screen exposure has been associated with lower cognitive abilities and academic performance in later years. Language development is also affected by screen time, as it diminishes the quantity and quality of interactions between children and caregivers. Contextual factors such as co-viewing and appropriateness of content play a role in determining the impact on language development. Excessive screen usage can also lead to problems in social-emotional development, including obesity, sleep disturbances, depression, and anxiety. It can impair emotional comprehension, promote aggressive behavior, and hinder social and emotional competence.

Parents play a crucial role in managing and reducing screen time by raising awareness, setting boundaries, and providing behavioral controls. Parental limitations and the absence of screens in bedrooms have been found to significantly reduce screen usage. Parents should also set an example by managing their own screen time. Overall, it is important for caregivers, educators, and healthcare professionals to understand the potential risks of excessive screen usage and implement strategies to promote healthy development in children, including alternative activities that foster cognitive, linguistic, and social-emotional skills.
